# On-chip combined radiotherapy and chemotherapy testing on soft-tissue sarcoma spheroids to study cell death using flow cytometry and clonogenic assay

**DOI:** 10.1038/s41598-019-38666-9

**Published:** 2019-02-18

**Authors:** Bishnubrata Patra, Julie Lafontaine, Maeva Bavoux, Karim Zerouali, Audrey Glory, Mohsen Ahanj, Jean-François Carrier, Thomas Gervais, Philip Wong

**Affiliations:** 1 Institute of Biomedical Engineering, École Polytechnique de Montréal, Montréal, Qc Canada; 2 Department of Engineering Physics, École Polytechnique de Montréal, Montréal, Qc Canada; 3 Institut du Cancer de Montréal, Montréal, Qc Canada; 4 Department of Pharmacology and Physiology, Université de Montréal, Montréal, Qc Canada; 5 Centre de Recherche du Centre Hospitalier de l’Université de Montréal (CRCHUM), Montréal, Qc Canada; 6 Department of Radiation Oncology, Centre Hospitalier de l’Université de Montréal (CHUM), Montréal, Qc Canada

## Abstract

Radiotherapy (RT) and chemotherapy (CT) are the major therapeutics to treat cancer patients. Conventional *in vitro* 2D models are insufficient to study the combined effects of RT and CT towards optimized dose selection or drug screening. Soft-tissue sarcomas (STS) are rare cancers with profound social impacts as they affect patients of all ages. We developed a microfluidic device to form and culture STS spheroids to study the combined cytotoxicities of RT and CT. Uniformly-sized spheroids of two different cell lines, STS 93 and STS 117, were formed in the device. RT doses of 0.5 Gy, 2 Gy, and 8 Gy were used in combination with CT, doxorubicin at 2 µM and 20 µM. The spheroids culture chambers within the device were arranged in a 3 × 5 matrix form. The device was made “peelable”, which enabled us to collect spheroids from each treatment condition separately. Collected spheroids were dissociated into single cells and evaluated using flow cytometry and clonogenic assays. Through this workflow, we observed that STS 93 spheroids treated with doxorubicin die through apoptosis, whereas RT induced death through other pathways. Spheroids from the p53 mutant STS 117 cell line were more resistant to RT and doxorubicin. The developed device could be used for the discovery of new drugs and RT synergies.

## Introduction

Cancer is a leading cause of death worldwide^1^. Surgery, radiotherapy (RT), and chemotherapy (CT) are the mainstay treatments for cancer patients. Although surgical removal of the tumor is often essential to cure many solid tumors, local recurrence rates remain high, even when negative surgical margins are obtained^2^. RT and CT are often administered prior to or after surgery to reduce the chance of local and metastatic recurrences. RT uses high energy electromagnetic waves, such as ionizing radiation (gamma rays), which upon interaction and ionization of intracellular water molecules, induce single and double stranded DNA breaks^3^. As a consequence to DNA damages, cells undergo a variety of DNA damage repair mechanisms and death pathways, which include apoptosis, necrosis, mitotic catastrophe and senescence^4^. CT are systemic agents which conventionally induce cell death or indefinite proliferative arrest that impedes cancer cells from regenerating a tumor^5^. Despite the clinically proven efficacy of RT and CT, they both induce substantial side effects to patients during and sometimes long after the completion of the treatments^6,7^.

Soft-tissue sarcomas (STS) are cancers that affect patients of any age and represent approximately 5% of pediatric and young adolescent cancers^8^. Standard treatment of patients with STS consists of surgery and adjunctive RT. The addition of radiosensitizing or radioprotective agents during RT could increase the efficacy of RT in killing cancer cells or reduce the long-term side effects of RT, respectively^9–12^. The use of adjunctive CT is controversial as a pooled analysis of two Phase III randomized clinical trials evaluating the use of doxorubicin-based CT to observation did not reveal an improvement in patient overall survival despite a reduction in relapses^13^.

Two-dimensional (2D) *in vitro* cancer models are potentially too simplistic and insufficient to accurately gauge the value of various combinations between RT and molecular agents. Three-dimensional (3D) *in vitro* models such as spheroids possess characteristics including close cell-cell interactions, lactic acidosis and hypoxia that could better mimic *in vivo* conditions and improve the screening accuracy for novel anti-cancer strategies^14–18^. A spheroid is a self-aggregation of cells without any matrix or physical support. As the size of spheroids increases, deeper lying cells may be exposed to increasing levels of lactic acid and subjected to hypoxia, which reduces the efficacy of RT. Similarly, certain drugs have difficulty diffusing and penetrating to the center of spheroids; hence the measured efficacy of RT and CT in 3D models are less than in 2D models^19,20^. Thus, the screen of combinatorial therapeutic agents for use with RT may yield candidates with higher subsequent developmental success rates when 3D spheroid models are used instead of or to complement monolayer models.

3D models within microfluidic devices are tools that exploit the manufacturing microchannels to miniaturize experiments to fit onto credit card-sized chips, thereby reducing reagent use, personnel time and experimental costs. Several groups have already used microfluidic devices to study the effect of anti-cancer drugs on spheroids^15,21,22^. Carr *et al*. used a microfluidic device to study RT-induced cell death in head and neck squamous cell carcinoma tissue biopsies through the quantification of soluble markers of cell death (lactate dehydrogenase assay and cytochrome c) and immunohistochemical assays^23^. Despite the potential advantages of spheroids as a model, it also has certain drawbacks. First, not all cell lines are able to form and be maintained as spheroids, thereby reducing the number of models. Second, 3D models confer heterogeneity in the concentrations of nutrients, oxygen and lactate within the spheroids, which are dependent on diffusion and therefore spheroid size^24,25^. Thus, consistent control of spheroid size is desired to reduce variability in experimental results. To our knowledge, there has not been an evaluation of microfluidic devices to assess combinations of RT and CT in STS spheroids.

**Figure 1:**
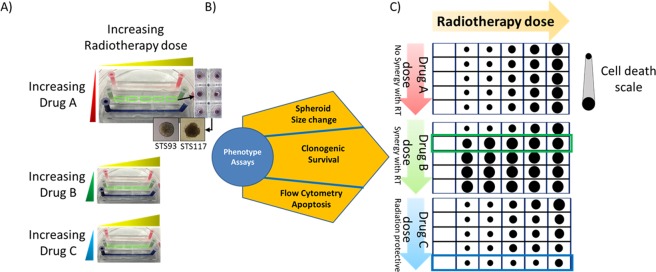
Diagram of strategy: (**A**) The polydimethylsiloxane (PDMS) microfluidic device used for radiotherapy (RT)/chemotherapy (CT) screening consists of 3 rows of 5 interconnected chambers, each containing two dozen spheroids grown in passivated wells. (**B**) Spheroids phenotyping under RT and drug exposure was performed using size change (microscopy), clonogenic survival (clonogenic assays), and flow cytometry (apoptosis). (**C**) Combinatorial response matrices CT-RT for synergy assessment. Hypothetical results of cell death from a drug with no effect on RT (top), RT/CT synergy (middle) and anti-synergy (radioprotective effect, bottom).

We developed a microfluidic platform to study the cytotoxic and anti-proliferative effects of RT and CT (doxorubicin) on STS spheroids. Spheroids were grown on chip and were then exposed to different doses of RT and CT on the same chip. The post-treatment phenotypes of the spheroids were observed and cell death was assessed (Fig. 1). Our proof of concept experiments suggest that this device could be used for the screening of radiosensitizing and radioprotective agents.

## Results

### Microfluidic spheroid formation

**Figure 2:**
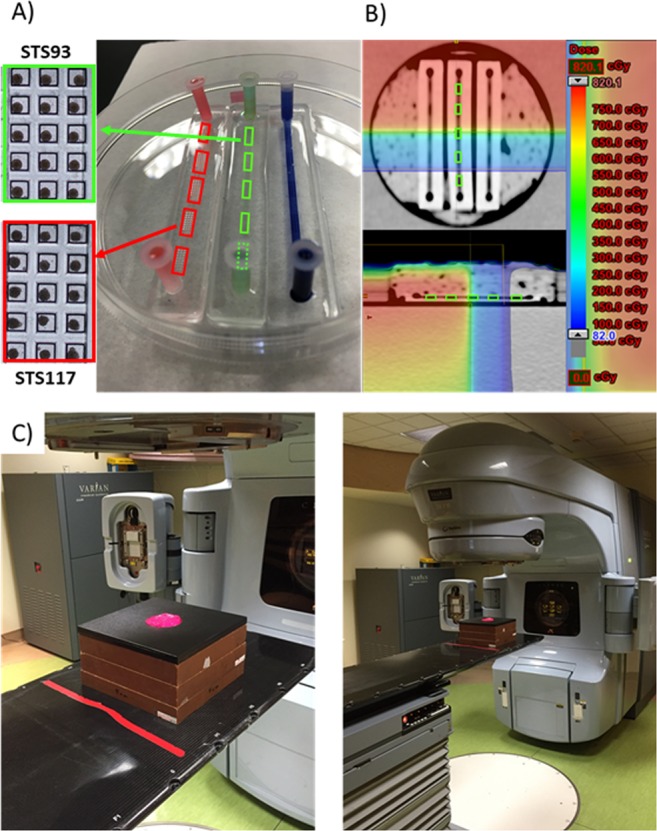
Experimental set-up. Within a 100 mm petri dish, the microfluidic systems are kept in sterile conditions (**A**). Each spheroid culture well is of the dimension 500 × 500 × 500 µm^3^. Two dozen (24) spheroid wells compose each of the 5 chambers (green and red rectangles), which are linearly connected within a single device. A CT-scan of the devices was done and a radiation dosimetry plan was generated (**B**) to treat different chambers within the devices with various doses of radiation (color coded) ranging from 0.5 Gy-8 Gy. The device and petri dish is covered by a pink bolus material (**C**, left), placed and irradiated using a clinical linear accelerator (**C**, right) as per the radiation plan. Spheroids of STS 93 and STS 117 were formed using the system within (**A**) 48 and (**B**) 120 hours following the introduction of a cell suspension of 2 × 10^6^ cells in 1 ml culture medium.

**Figure 3:**
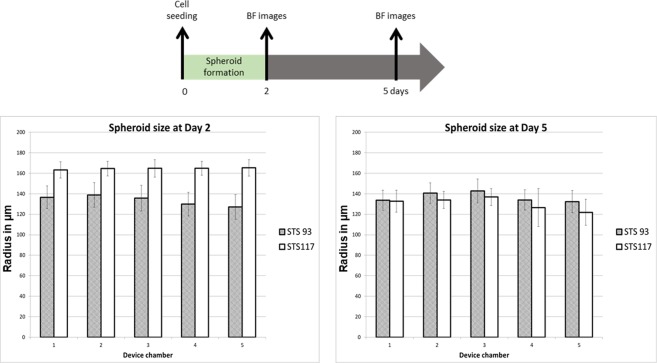
Spheroid formation and size. Size (mean and standard deviation) distribution of the spheroids cultured in different systems were examined using bright field (BF) imaging. Spheroids of STS 93 and STS 117 were formed using the system within 48 and 120 hours following the introduction of a cell suspension of 1 × 10^6^ cells in 1 ml culture medium through the inlet. Each spheroid culture chamber is of the dimension 500 × 500 × 500 µm^3^. The timeline of the experiments is depicted above the results.

Within 48 hours of loading 1 × 10^6^/mL single cells into the devices, STS 93 and STS 117 spheroids were formed. Representative images of device chambers containing STS 93 and STS 117 spheroids are shown in Fig. 2A. Of the 360 spheroids analyzed, the mean radius of STS 93 spheroids measured 132 µm (Standard Deviation (SD): 17 µm) (Fig. 3). The mean radius of the STS 117 spheroids measured 164 µm (SD: 17 µm) (Fig. 3). Five days following the single cells loading, the mean STS 93 and STS 117 spheroid radius measured 137 µm (SD: 11 µm) and 153 µm (SD: 16 µm), respectively (Fig. 3).

### Radiotherapy dose simulation

Figure 2B shows the RT dose plan for the treatment of the systems. While the RT method used in our experiments can administer different RT to different chambers within the same device, electron scattering secondary to the photons interacting with the irradiated materials limits (1) the minimum dose received by a chamber, (2) the RT dose homogeneity within a chamber and (3) the dose escalation rate from one chamber to another. The calculated mean dose received by chamber 1 was 0.34 Gy (range: 0.29–0.42 Gy), henceforth spheroids receiving this RT dose range will be referred as 0.5 Gy in subsequent text and figures. Contents within chamber 2 received a mean dose of 2.0 Gy (range: 1.9–2.1 Gy). To increase the dose homogeneity within Chamber 4 and 5, we used the space of Chamber 3 to ramp up the RT dose. Chamber 4 and 5 received a mean RT dose of 7.8 (Range: 7.8–7.9 Gy) and 8.0 Gy (8.0–8.0 Gy), respectively.

### Acute effects of treatments

**Figure 4:**
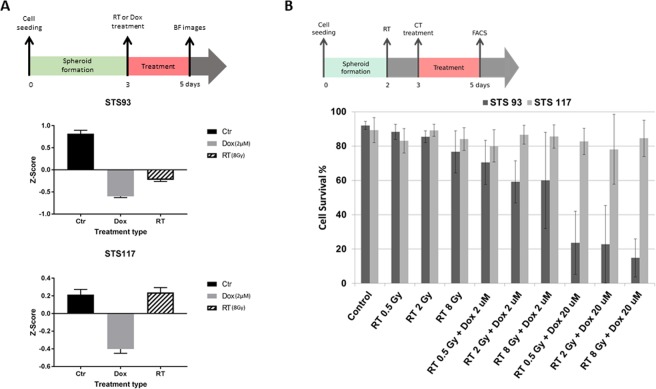
Treatment response to radiation and doxorubicin. (**A**) Bright field (BF) imaging and measurement of spheroid size 48 hours after the treatment with control, radiation (RT 8 Gy) and doxorubicin (Dox 2 µM). Spheroid size were converted into Z-scores to allow for comparisons between experiments using various seeding concentrations of cells (1 × 10^6^ or 2 × 10^6^ cells/ml). Data represent the means ± standard error of the means (n = 133–270). Size differences were compared using paired student t-tests. Spheroid cell viability at 48 hours was assessed by (**B**) flow cytometry analyses in which surviving cells consisted of cells not considered apoptotic or necrotic. The timeline of each set of experiments are depicted above each (**A**,**B**) result figures.

Using bright field (BF) microscopy to follow spheroid sizes before and after treatments, we observed that, compared to STS 93 spheroids irradiated with 0–0.5 Gy (N = 188), there was a significant (p < 0.0001) reduction in radius of the spheroids that received 8 Gy (N = 232) of RT (Fig. 4B). In contrast, STS 117 spheroids that received 8 Gy (N = 343) of RT were similar (p = 0.9) to those that received 0–0.5 Gy (N = 332) (Fig. 4B). Doxorubicin (2 µM) induced significant (p < 0.0001) reduction in spheroid (STS 93 (N = 213) and STS 117 (N = 346)) size within 72 hours of the treatment as compared to spheroids that received 0–0.5 Gy.

Flow cytometry of dissociated spheroid cells observed a high proportion (>80%) of survival in STS 93 and STS 117 treated under control conditions 5 days after cell seeding. RT 0.5 Gy,2 Gy and 8 Gy did not induce a significant amount of apoptosis or necrosis in the cells (Fig. 4C, Table 1). An increasing dose of doxorubicin was associated (Pearson correlation p < 0.001) with an increasing amount of early STS 93 cell deaths. No significant acute cell death effect from doxorubicin or RT was observed in STS 117 cells. As flow cytometry evaluates cell deaths (apoptosis and necrosis) 48 hours following CT and/or RT, surviving cells may still undergo subsequent cell death or proliferative arrest, which are encompassed by clonogenic assays.

**Table 1:** Comparison of flow cytometry STS 93 and STS 117 cell survival at 48 hours after treatments.

STS 93	RT 0.5 Gy	RT 2 Gy	RT 8 Gy	RT 0.5 Gy + Dox 2 µM	RT 2 Gy + Dox 2 µM	RT 8 Gy + Dox 2 µM	RT 0.5 Gy + Dox 20 µM	RT 2 Gy + Dox 20 µM	RT 8 Gy + Dox 20 µM
Ctr	0.2	0.05	0.1	0.05	0.01	0.1	**0.003**	**0.006**	**0.0003**
RT 0.5 Gy		0.4	0.2	0.09	0.02	0.2	**0.004**	**0.008**	**0.0004**
RT 2 Gy			0.3	0.1	0.02	0.2	**0.004**	**0.009**	**0.0005**
RT 8 Gy				0.6	0.2	0.4	0.01	0.02	**0.003**
RT 0.5 Gy + Dox 2 µM					0.3	0.6	0.02	0.03	**0.005**
RT 2 Gy + Dox 2 µM						1	0.05	0.07	0.01
RT 8 Gy + Dox 2 µM							0.1	0.1	0.06
RT 0.5 Gy + Dox 20 µM								1	0.5
RT 2 Gy + Dox 20 µM									0.6
**STS 117**	**RT 0.5 Gy**	**RT 2 Gy**	**RT 8 Gy**	**RT 0.5 Gy + Dox 2 µM**	**RT 2 Gy + Dox 2 µM**	**RT 8 Gy + Dox 2 µM**	**RT 0.5 Gy + Dox 20 µM**	**RT 2 Gy + Dox 20 µM**	**RT 8 Gy + Dox 20 µM**
Ctr	0.4	1	0.4	0.2	0.6	0.6	0.3	0.4	0.6
RT 0.5 Gy		0.2	0.8	0.7	0.5	0.7	1	0.7	0.8
RT 2 Gy			0.3	0.2	0.5	0.4	0.2	0.4	0.5
RT 8 Gy				0.5	0.6	0.8	0.8	0.6	1
RT 0.5 Gy + Dox 2 µM					0.4	0.4	0.7	0.8	0.6
RT 2 Gy + Dox 2 µM						0.9	0.7	0.6	0.8
RT 8 Gy + Dox 2 µM							0.7	0.6	0.9
RT 0.5 Gy + Dox 20 µM								0.7	0.8
RT 2 Gy + Dox 20 µM									0.6

Treatments consisted of radiotherapy (RT) given at 0.5 Gy, 2 Gy or 8 Gy, or doxorubicin (Dox) at 2 µM or 20 µM. Control (Ctr) conditions were not irradiated or treated with doxorubicin. Student’s t-test p values for each comparison are indicated in the table. Populations with significant (p < 0.006) differences in cellular survival are in bold.

### Clonogenic assay analysis of cumulative treatment toxicity

**Figure 5:**
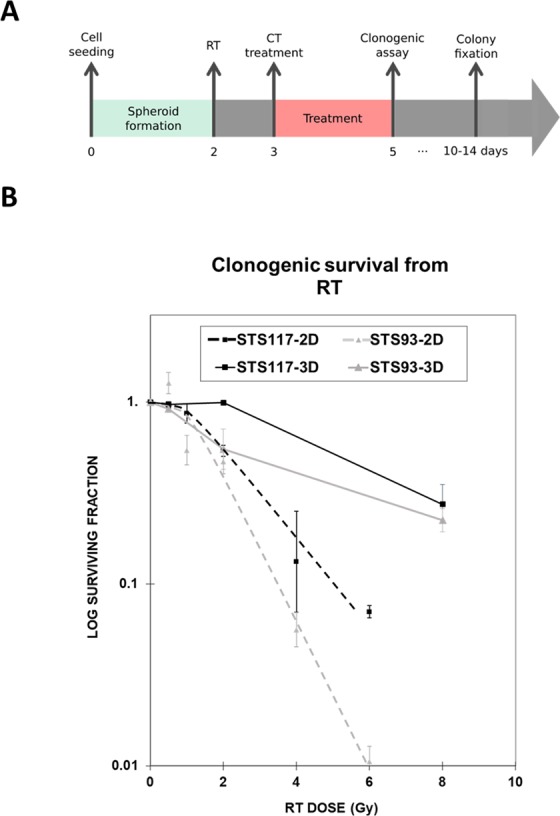
Analysis of cumulative cell survival from RT. (**A**) Experimental timeline for spheroids. (**B**) Clonogenic survival of STS 93 and STS 117 monolayer cells (2D) or spheroids (3D) treated with 0, 0.5, 2, 6 and 8 Gy of radiation (RT). Cell survival (mean ± standard deviation) is normalized to the clonogenic formation from cells treated at 0 Gy (n = 3). The initial seeding concentration for spheroid formation within the device was 2 × 10^6^ cells/ml.

The clonogenic assay measures the cumulative toxicities secondary to all cell death pathways including necrosis, apoptosis, mitotic catastrophe, and senescence from RT and CT. A dose dependent clonogenic cell death was observed in both STS 93 and STS 117 cells following RT. When the cells were treated in spheroids (3D), STS 93 cells were more sensitive than STS 117 cells. Their relative sensitivity to RT remains consistent with when the cells were treated in monolayer (Fig. 5). RT 8 Gy induced 78% and 73% of cell death in STS 93 and STS 117, respectively. Spheroids treated with supratherapeutic doxorubicin (2 µM or 20 µM) failed to form colonies, suggesting that the surviving cells as detected in flow cytometry (Fig. 4C) subsequently continue to die.

## Discussion

The management of cancer patients often involves RT and CT. Although the use of cancer spheroid or organoid models are increasingly being employed to screen systemic agents^26^, their application in RT^23,27^ and the combination of RT and CT^28,29^ is less described. Our microfluidic device provides a promising platform to evaluate the interaction between RT and CT on *in vitro* spheroid models. Uniformly sized spheroids of different cell lines were formed and cultured within the device with little manipulations. One hundred and twenty spheroids are formed within the 5 chambers in the device, allowing delivery of different RT doses to spheroids incubated within the same CT conditions. Furthermore, spheroids from different chambers could be collected separately for additionnal assessments.

Clonogenic assays represent the gold standard method in quantifying cell deaths and proliferative loss from all cellular pathways in reaction to injuries and treatments. The relative sensitivity of STS 93 and STS 117 clonogenic cell death secondary to RT treatment was maintained when they were irradiated as spheroids. However, RT induced less toxicities to cells in spheroids than in monolayer, which is consistent with previously published results evaluating CT^19,26,30^. Unlike in CT where drug penetration into spheroids reduces its efficacy, the reduction in RT efficacy in spheroids may be secondary to (1) close cell-cell interactions and (2) limitation in oxygen diffusing into the center of spheroids, thereby producing a hypoxic center, a known characteristic in most solid tumor that reduces RT efficacy^19^. Thus, the current model may better recapitulate physiological tumor microenvironment to provide a more accurate measure of treatment effects when combined with RT.

It is well known that p53 is an important regulator of cell fate following DNA damages. Specifically, in the absence of functional p53, cellular apoptosis secondary to irradiation is reduced. Similarly, the rate of apoptosis in STS 117 (p53 loss of function mutant) cells following RT was less than in STS 93 (wild-type p53). As apoptosis represents early cell death, often occurring within hours to days following the injury, we correspondingly observed a significant reduction in spheroid size 2 days after RT in STS 93 cells treated with 8 Gy. The size of RT treated STS 117 spheroids was similar to the size of control STS 117 spheroids after 2 days, suggesting that STS 117 do not undergo early cell death such as apoptosis. This is consistent with the fact that STS 117 cells harbor a mutated p53 therefore they are more resistant to apoptosis than p53 wild-type cells.

The cell line specific resistance to apoptosis was similarly observed when spheroids were treated with doxorubicin. Cell suspensions obtained from STS 93 spheroids treated with doxorubicin were going through apoptosis in a doxorubicin dose dependent manner. Similarly, a doxorubicin dose dependent reduction in spheroid size was observed by day 2 post-RT. Akin to treatments by RT, STS 117 cells treated with doxorubicin were not undergoing more apoptosis than spheroids in control condition. However, their spheroid sizes reduced following the treatments, suggesting that STS 117 cells were undergoing cell deaths through means other than senescence or apoptosis. Our results suggest that change in spheroid size may be an indicator of the living cell population within the spheroids. Nevertheless, as shown by clonogenic and flow cytometry assays, apoptotic cell death represents a minority of cell death from RT. As we were unable to grow clones from spheroids treated by supratherapeutic doxorubicin (2 µM or 20 µM), it is likely that other types of cell death beyond apoptosis also occur following CT. As the maximum plasma concentration obtained during clinical use of doxorubicin is <1 µM, the evaluation at 2 µM or 20 µM mainly served as proof-of-concept experiments to demonstrate the observable changes that could be detected in spheroids secondary to a potent apoptosis inducer that is also regulated by p53.

## Conclusion

We developed a microfluidic device to study the combined effect of RT and CT using a 3D *in vitro* spheroid model. A clinical RT simulation, planning and treatment platform was used to administer differential doses of RT to the microfluidic devices. The cytotoxic effects of RT administered using this clinical platform recapitulated the relative cellular sensitivity to RT on monolayer cultures treated using a laboratory irradiator. This strategy therefore can be exploited to augment the result output from the use of microfluidic devices that can be simultaneously treated with different RT doses and molecular agents. Importantly, spheroids from each chamber can subsequently be imaged within the translucent devices or be isolated independently for further evaluations. Through our proof-of-concept assays, we could demonstrate the genomic phenotype of STS 117 in its resistance to undergo apoptosis following RT and CT. We also noted a correlation between the quantity of apoptotic cells and a reduction in spheroid size following RT and CT. As apoptosis only represent a portion of RT or CT induced cell deaths, clonogenic assays still need to be used to verify the efficacy of treatments and their combinations. Ongoing work in evaluating novel and more efficient techniques to reproduce the results of clonogenic assays is being conducted to increase the throughput of the platform for use in drug screening and radiosensitizer discovery.

## Materials and Methods

### Microfluidic Devices

Microfluidic systems were made of polydimethylsiloxane (PDMS). For the purpose of experimental flexibility, we constructed individual devices that had 5 linearly connected chambers, each containing 24 uniformly sized 125 nL cubic traps for spheroid formation (Fig. 2A). The dimension of each device is about 7.5 × 1 cm^2^. The microfluidic device layout was designed using CATIA (Dassault Systemes, France), based on previously published design rules for spheroid culture chips to ensure proper oxygenation and nutrient supply^31^. The mold was fabricated on poly-methyl methacrylate (PMMA) using micromachining. The device is made of two layers of PDMS: the bottom layer contains spheroid culture chambers with dimensions 500 × 500 × 500 µm^3^, and the top layer contains a straight channel covering all the culture chambers. Each device could be treated with a particular systemic agent, while each chamber could be treated with a particular RT dose (Fig. 2B).

### Cell culture and spheroid formation

Two primary human STS cell lines were used (STS 93: harboring a wild type TP53 and STS 117: harboring a loss of function mutation TP53), which were previously described^32^. Briefly, these cell lines were derived from the patients’ primary extremity STS, which were pathologically diagnosed as undifferentiated pleomorphic sarcomas, a high grade STS with a high propensity to recur locally and distantly as metastases. STS cells were kindly provided by Dr. R. Gladdy (Mount Sinai Hospital, ON, Canada). Cells were incubated at 37 °C under 5% CO_2_ in DMEM:F12 1:1 media (Thermo Fisher, ON, Canada) with 10% bovine serum (Sigma-Aldrich, ON, Canada), and 1% pen-strep (Thermo Fisher, ON, Canada). Cells dissociation was done using 0.25% trypsin-ETDA (Wisent, QC, Canada).

Prior to cell seeding, the microfluidic devices were cleaned with 70% EtOH, treated with 10% Pluronic F-108 for two hours, cleaned again with 70% EtOH and rinsed with HBSS twice. Finally, the fluid within the systems was replaced by fresh culture medium. A cell suspension of 1-2 × 10^6^ cells in 1 ml culture medium was introduced at the inlet, and 100 µl culture medium was taken out from the outlet. Then, for the same device, the cell suspension was introduced from the outlet and 100 µl culture medium was taken out from the inlet. This process was repeated 3 times from both directions (inlet/outlet) to improve the homogeneity in cellular concentration across the device chambers. The device was then kept in an incubator. Cell medium was changed every 48 hours with 300 µl of fresh culture medium.

### Radiotherapy and Chemotherapy treatments

To derive a RT plan that would give 3 different RT doses (0.5 Gy, 2 Gy and 8 Gy) to different chambers within the devices, three empty devices were housed in a phantom and scanned by computed tomography (CT). The phantom consisted of two layers of solid 10 cm slabs with water-like electron densities, which sandwiched the petri dish that contained the 3 devices surrounded by water-like bolus material to reduce RT backscattering. The CT images were used for RT dosimetric calculations and planning on the Eclipse Treatment Planning System (Varian Medical System, Palo Alto, CA). The Eclipse Treatment Planning System is a commercial RT software approved by FDA for clinical use. Subsequently, spheroid-containing devices were irradiated using a linear accelerator commissioned for clinical use (Clinac 21EX, Varian Medical System, Palo Alto, Ca). RT was delivered 48 hours following cell seeding in the devices.

Doxorubicin (Sigma-Aldrich, ON, Canada) is a chemotherapy used in the treatment of cancer, including STS^33–35^. Doxorubicin was dissolved in normal cell culture medium to yield concentrations of 2 µM and 20 µM for the treatment of STS spheroids. Cell culture medium within the devices was replaced with doxorubicin containing medium 24 hours after RT. Spheroids were incubated in doxorubicin containing medium for 48 hours prior to cell death analyses (72 hours after RT or day 5). Monolayer cells were treated using the same conditions as described for spheroids. Cells on petri dish were treated at 70–80% confluence with RT (Gammacell irradiator).

### Microscopy imaging and image analysis

Spheroid uniformity and size were measured using images obtained through an inverted microscope (Nikon TS100) with a 4x objective with a 0.1 numerical aperture (NA). The radius of the spheroids were estimated using ImageJ (v. 1.49, National Institute of Health, Bethesda, MD). The radius of the spheroids from individual experiments were normalized to derive Z-scores using the following formula:

$$z=\frac{x-\mu }{\sigma }$$

where: χ is the raw radius of the spheroids. µ is the mean radius of the spheroids within an experiment. σ is the standard deviation of the spheroid radius within an experiment.

### Flow cytometry analysis and clonogenic assay

Post-treatment spheroids were collected from the devices and segregated according to each treatment condition. Single cell suspensions from the treated spheroids were obtained through trypsinization (37 °C for 15 minutes). Half of the single cells were stained for Annexin V (Alexa Fluor® 488 conjugated, Thermo Fischer Scientific, ON, Canada) according to the manufacturer’s recommended conditions and DAPI (Sigma-Aldrich, CA). Cells were immediately counted by flow cytometry (Fortessa, BD Biosciences, NJ) and analyses were performed using FlowJo (Oregon, USA).

The rest of the single cell suspensions were used for clonogenic assays in which cells from each condition were seeded in triplicates in 12-well plates (Nunc, Thermosfisher Scientific, ON, Canada). The plates were left in the incubator for 10–14 days to allow for colony formations. Subsequently, the colonies were fixed and stained in 70% methanol solution containing 0.5% Crystal Violet (Sigma-Aldrich, CA). Colonies were counted and normalized with respect to the control condition.

## Data Availability

All data generated or analyzed during this study are included in this published article.
